# Retraction

**DOI:** 10.1002/cam4.5090

**Published:** 2022-08-17

Xiang, W, Lv, L, Zhou, G, et al. The lncRNA SNHG5‐mediated miR‐205‐5p downregulation contributes to the progression of clear cell renal cell carcinoma by targeting ZEB1. Cancer Med. 2020; 9: 4251–4264. https://doi.org/10.1002/cam4.3052

The above article, published online on April 12, 2020 in Wiley Online Library (wileyonlinelibrary.com), has been retracted by agreement between the authors, the journal's Editor in Chief, Dr Stephen Tait, and John Wiley & Sons Ltd. The retraction has been agreed following concerns raised by a third party regarding duplicated panels in figures within the article. Following the concerns raised, an investigation by the journal team found that there were discrepancies between the raw data and published images across several figures, including 6B, 2E, 5B, 3D, 5D, and 5F. The authors were unable to provide a satisfactory explanation for these discrepancies. The authors and the editors no longer have confidence in the results and conclusions.

